# Can financial payments incentivize short-term smoking cessation in orthopaedic trauma patients? Evidence from a discrete choice experiment

**DOI:** 10.1186/s13561-021-00313-3

**Published:** 2021-04-26

**Authors:** Dana Alkhoury, Jared Atchison, Antonio J. Trujillo, Kimberly Oslin, Katherine P. Frey, Robert V. O’Toole, Renan C. Castillo, Nathan N. O’Hara

**Affiliations:** 1 Department of Health Policy and Management, Johns Hopkins Bloomberg School of Public Health, Baltimore, MD USA; 2 Department of Orthopaedics, University of Maryland School of Medicine, 110 South Paca St., Suite 300, Baltimore, MD USA; 3 Department of International Health, Johns Hopkins Bloomberg School of Public Health, Baltimore, MD USA

## Abstract

**Background:**

Smoking increases the risk of complications and related costs after an orthopaedic fracture. Research in other populations suggests that a one-time payment may incentivize smoking cessation. However, little is known on fracture patients’ willingness to accept financial incentives to stop smoking; and the level of incentive required to motivate smoking cessation in this population. This study aimed to estimate the financial threshold required to motivate fracture patients to stop smoking after injury.

**Methods:**

This cross-sectional study utilized a discrete choice experiment (DCE) to elicit patient preferences towards financial incentives and reduced complications associated with smoking cessation. We presented participants with 12 hypothetical options with several attributes with varying levels. The respondents’ data was used to determine the utility of each attribute level and the relative importance associated with each attribute.

**Results:**

Of the 130 enrolled patients, 79% reported an interest in quitting smoking. We estimated the financial incentive to be of greater relative importance (ri) (45%) than any of the included clinical benefits of smoking cessations (deep infection (ri: 24%), bone healing complications (ri: 19%), and superficial infections (ri: 12%)). A one-time payment of $800 provided the greatest utility to the respondents (0.64, 95% CI: 0.36 to 0.93), surpassing the utility associated with a single $1000 financial incentive (0.36, 95% CI: 0.18 to 0.55).

**Conclusions:**

Financial incentives may be an effective tool to promote smoking cessation in the orthopaedic trauma population. The findings of this study define optimal payment thresholds for smoking cessation programs.

## Background

Smoking is associated with a wide range of complications in the orthopaedic trauma population [1–6]. Specifically, previous studies suggest that smoking and tobacco use may be associated with an increased risk of bone healing complications and infections [7–20]. Fortunately, the physiological effects of smoking and tobacco use observed after a fracture have been shown to be reversible within a short time frame. Smoking cessation during the early recovery phase can decrease the risk of these complications considerably, as both wound healing and the immune system improve within 6 weeks following cessation [21–23]. In three prospective randomized trials, perioperative smoking cessation demonstrated a 50% reduction in the risk of complications among successful quitters [24–26]. These findings may be particularly important in the orthopaedic trauma population, as complications frequently occur relatively within a few months after surgery.

Traditional smoking cessation interventions have demonstrated limited long-term success resulting in a quit rate of 5–10%, with only 25% of smokers even utilizing the suggested programs [27, 28]. However, multi-modal interventions have shown a roughly doubled quit rate than single modality programs. Most combined interventions include various combinations of educational, behavioral, and pharmacologic components [29, 30].

Prior research suggests that financial incentives may be effective for smoking and tobacco use cessation [31–37]. A recent randomized trial compared smokers who received a one-time payment of $750 with those who received smoking cessation information but no monetary incentive [38]. The trial demonstrated that cessation within the first 6 months nearly doubled, and enrollment in educational programs tripled with the addition of a financial incentive. In addition, the trial showed that financial incentives for smoking cessation significantly increased the rates of smoking cessation over 18 months. A 2019 systematic review suggests that financial incentives improve long-term smoking cessation rates in mixed population studies [39].

While these findings are critically important in the context of general health, in the orthopaedic trauma population, most smoking-related complications occur within the first year after injury [1, 2]. Consequently, financial incentive programs may be especially beneficial if implemented early in the healing process to reverse the harmful health conditions associated with smoking during recovery from an orthopaedic injury.

The objectives of this study were to determine the willingness of orthopaedic trauma patients to accept a financial incentive to stop smoking after their fracture and quantify the level of financial incentive and clinical benefits required to motivate smoking cessation. We hypothesized we would be able to model the utility of financial incentives and clinical benefits that would motivate patients to stop smoking after their fracture.

## Methods

### Study design

The study utilized a discrete choice experiment (DCE) to elicit patient preferences towards financial incentives and reduced complications associated with smoking cessation. DCEs are a well-established quantitative method that elicits stated preferences based on responses to described hypothetical scenarios. The response data are used to calculate the utility of each attribute level and the relative importance associated with each attribute [40–44]**.** Tradeoff estimates can also be calculated using the utility values. DCEs have been extensively used in the healthcare literature to elicit patient preferences [45, 46].

### Study setting and population

This cross-sectional study was performed at a single Level 1 trauma center. The study has received ethical approval (HP-00083110) from the Institutional Review Board at the University of Maryland. The study included adult patients with a surgically treated extremity fracture and self-identified as a smoker. We excluded patients who were unable to speak and read English. Patients were enrolled at their postoperative clinic visits from January through August 2019. The median time from injury to consent for the survey was 50 days (IQR: 21–157).

### Survey development

**Fig. 1:**
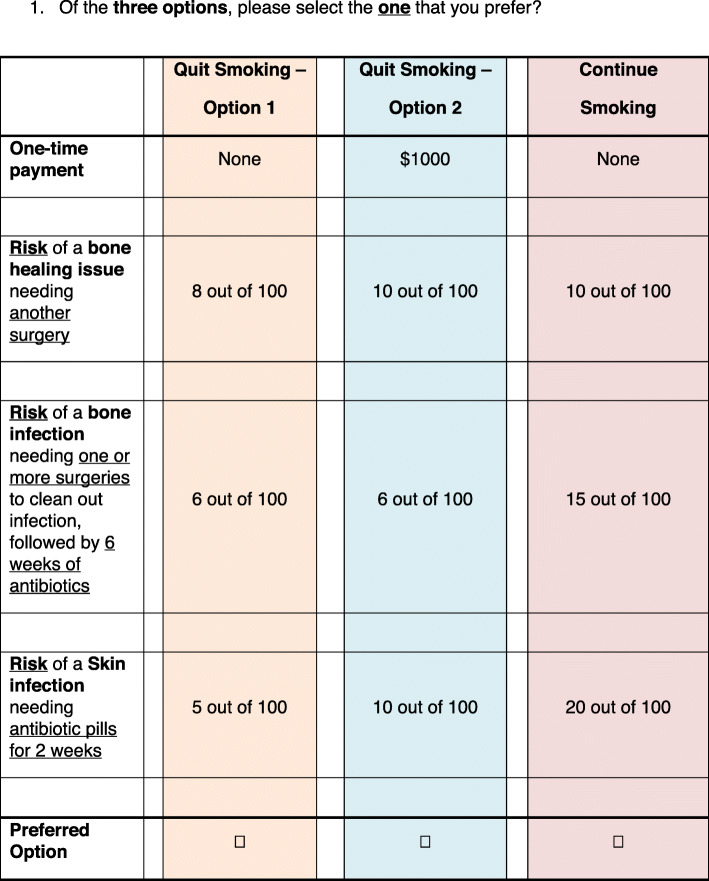
Sample choice set

The survey presented 12 sets of hypothetical options, called choice sets, in which two potential clinical outcomes and an associated financial incentive were described (Fig. 1). The options described in the choice sets included possible financial incentives ranging from $0 - $1000, and clinical benefits, which included various risk levels for a nonunion, deep surgical site infection (SSI), and superficial SSI. Each choice set also had a “status quo” option, which provided no financial incentive, a 10% risk of nonunion, a 15% risk of a deep SSI, and a 20% risk of a superficial SSI (Table 2 in Appendix ). The size of the rewards and frequency were informed by a recent randomized trial that studied financial incentives for smoking cessation in the general population [38]. The risk attributes and their levels were designed based on patients’ reported outcomes, consultation with orthopaedic trauma surgeons, and a literature review [7, 47–50]. The experiment design used a D-efficiency approach to maximize the orthogonality of the attributes and levels included in the 12 choice sets [51].

### Survey Administration

Consenting patients completed the paper-based DCE survey during a follow-up clinic visit. Prior to completing the survey, patients were informed that the objective of this study was to understand the willingness to quit smoking for a financial reward or a reduced risk of medical complications. The survey included a preamble describing the increased risk of a medical complication for smokers with injuries, as well as the recommendation that patients quit smoking to reduce their risk of these medical complications. The respondents were instructed that the hypothetical one-time payment would be provided if the patient abstained from smoking for 6 months after injury.

To ensure respondents understood the survey, we included an internal comprehension check in the design. One of the included choice sets had one option that was beneficial to the patient in all dimensions (financial incentive and postoperative complications). Selecting the inferior option for this choice set signified a failure to comprehend the survey, and the respondent’s data were not included in the final analyses.

Demographic data included age, sex, race, education, income, health insurance status, and socioeconomic deprivation, as measured by the Area Deprivation Index (ADI). The ADI was developed by the Health Resources and Services Administration and represents a geographic area-based measure of the socioeconomic deprivation experienced by a neighborhood as a composite score of 17 variables, including income disparity and percent of the population age 25 and older with at least a high school diploma [52]. The survey also included questions on the participant’s interest in quitting smoking and the available level of social support. We used these questions to explore heterogeneity in responses to financial rewards to smoking cessation.

### Statistical analysis

There is no consensus on a sample size calculation for discrete choice experiments. However, previous research recommends 50 participants per subgroup in the analysis [40–42]. Based on this heuristic, 100 respondents would provide adequate power to model a binary subgroup.

The patient characteristics were described using counts with proportions for categorical data and means with standard deviations or medians with interquartile ranges for continuous variables, depending on the distribution. The DCE response data were effects coded and analyzed using a multinomial logit model [53]. We calculated each attribute’s relative importance by dividing the LogWorth (i.e., −log_10_ multiplied by the *p*-value of the likelihood ratio test) of the attribute by the sum of the LogWorths of the attributes included in the model [54, 55]. The model parameter estimates denote the mean utility for a given level within each attribute. Willingness to pay values were used to determine the acceptable tradeoffs between a reduced financial incentive and the reduced risk of a postoperative complication. We assessed preference heterogeneity by adding covariates as interaction terms into the model. The covariates that we hypothesized to be associated with differential preferences included sex, high school education or less, an income less than the poverty level, and living in an area of high deprivation (ADI of 8 or higher). Interactions of *p* < 0.05 were considered significant. We did not adjust our Type I error for the testing of multiple subgroups, and these results should be considered exploratory. Missing data were not imputed and were assumed to be missing completely at random. The analyses were performed with JMP Pro Version 14 (Cary, NC) and R Version 3.6.1 (Vienna, Austria).

## Results

### Patients

Of the 201 eligible patients, 130 (65%) consented to participate in the study and completed the survey. An additional 16% failed the survey’s internal comprehension check and were excluded from the analysis. Of the 109 patients included in the final analyses, the mean age was 40 years (SD: 13) and 66% (*n* = 72) of the respondents were male (Table 1). The median household income was $27,500 (IQR: $5000 - $62,500), and 38% of the respondents had a high school education or less. Approximately one-third of the respondents were insured by Medicaid, and two-thirds of the respondents lived in neighborhoods with high levels of deprivation. Seventy-nine percent of the sample reported being interested in quitting smoking. Sixteen respondents (15%) always selected the “neither” option, suggesting an unwillingness to quit smoking regardless of any benefit from a financial incentive or an increased risk of a postoperative complication. Fourteen respondents (13%) consistently selected the option with a higher payment.

**Table 1:** Patient characteristics (*n* = 109)

Age, mean (SD)		40.4 (13.0)
Sex, male, n (%)		72 (66)
Race, n (%)		
	White	55 (50)
	African-American	42 (39)
	Other	11 (10)
Educational attainment, n (%)^a^		
	High school or less	41 (38)
	Some college or degree	68 (62)
Household income, median (IQR)^b^		$27,500 ($5000 - $62,500)
Health insurance, n (%)		
	Medicaid	39 (36)
	Medicare	32 (29)
	Private employer-based	23 (21)
	Other public insurance	12 (11)
	Uninsured	3 (3)
Area deprivation index, n (%)^c^		
	Top quartile	17 (16)
	2nd quartile	18 (17)
	3rd quartile	35 (33)
	Lowest quartile	37 (35)
Interested in quitting, n (%)		86 (79)
Social/emotional support available, n (%)^d^		
	Always	46 (42)
	Usually	19 (17)
	Sometimes	27 (25)
	Rarely	10 (9)
	Never	4 (4)

Abbreviation: *IQR* Interquartile range

^a^ One participant refused to answer question

^b^ 25 participants refused to answer question

^c^ Two participants had out of state addresses

^d^ Three participants refused to answer question

### Relative importance

**Fig. 2:**
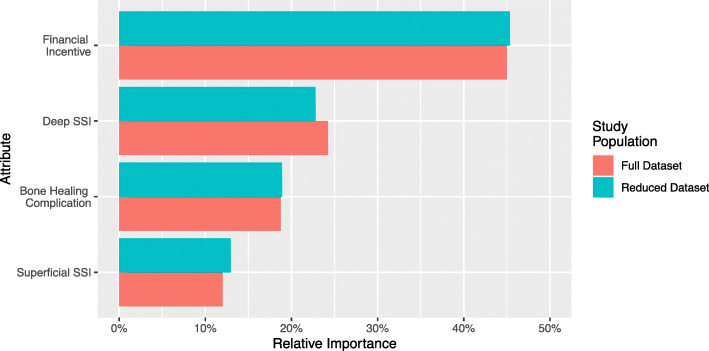
The relative importance of the included attributes calculated with the full sample and with a reduced sample that excluded patients that were unwilling to stop smoking based on any of the presented options. SSI, surgical site infection

Of the included attributes, a financial incentive had the greatest relative importance to the respondents (relative importance (ri): 45%) (Fig. 2). The risk of a deep infection ranked second (ri: 24%). Bone healing complications (ri: 19%) and superficial infections (ri: 12%) were of lesser importance. When we excluded patients who were unwilling to stop smoking based on any presented options, the relative importance of the included attributes did not qualitatively change.

### Mean utility of attributes levels

**Fig. 3:**
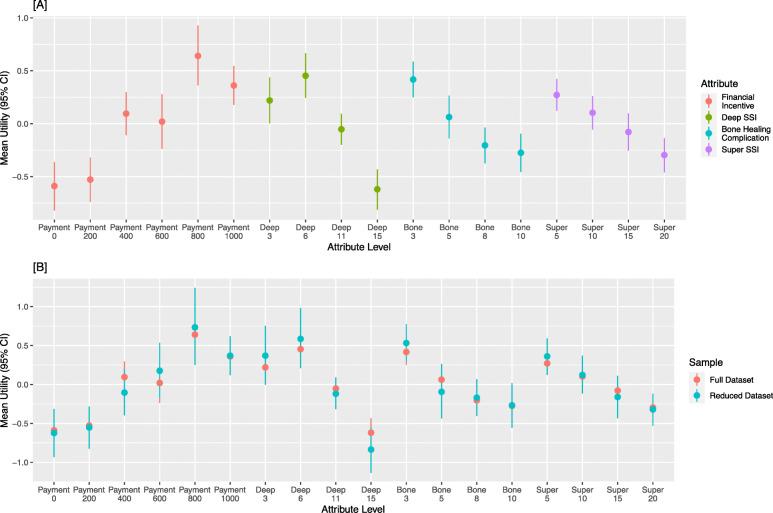
The mean utility with 95% confidence interval for each of the included attributes and levels. **a** Reports the full sample. **b** Reports both the full dataset and with a reduced sample that excluded patients that were unwilling to stop smoking based on any of the presented options. SSI, surgical site infection

A one-time payment of $800 provided the greatest utility to the respondents (0.64, 95% CI: 0.36 to 0.93), surpassing the utility associated with a single $1000 financial incentive (0.36, 95% CI: 0.18 to 0.55) (Fig. 3a). Both the financial incentives of $800 and $1000 provided substantially more utility than a one-time incentive of $600 or less. The gain in utility from no financial incentive to the $800 incentive (mean difference (md): 1.2, 95% CI: − 1.6 to − 0.9) was greater than the utility lost when one’s risk of a deep SSI (md: 1.1, 95% CI: − 1.4 to − 0.8), bone healing complication (md: 0.7, 95% CI: − 0.9 to − 0.4), or superficial SSI (md: -0.6, 95% CI: − 0.8 to − 0.3) declined within the scale included in the experiment. When we excluded patients who were unwilling to stop smoking based on any presented options, the effects were marginally increased (Fig. 3b).

### Willingness to pay

We used willingness to pay estimates to determine the value associated with a 1% decrease in the risk of a postoperative complication. Based on the respondents, patients would forgo $84 (95% CI: $56 to $112) of the financial incentive for a 1% decline in their risk of a bone healing complication. Similarly, respondents would forgo $77 (95% CI: $53 to $101) of the financial incentive for a 1% decline in their risk of a deep SSI and $36 (95% CI: $22 to $ 49) of the financial incentive for a 1% decline in their risk of a superficial SSI.

### Heterogeneity in preferences

We tested several covariates, including sex, education, income, and neighborhood deprivation, for possible heterogeneity in preferences. None of these covariates met the *p* < 0.05 threshold when added as interaction terms into the model.

## Discussion

Our findings suggest that an $800 one-time payment may adequately incentivize short-term smoking cessation in orthopaedic trauma patients. The benefits of the financial incentive were of greater importance to the respondents than the clinical benefits of smoking cessation. The preference for the financial incentive was homogenous across the sample.

It is possible that an $800 financial incentive was more desirable to patients than $1000 due to a loss in intrinsic motivation with a payout that exceeds a certain threshold. Behavioral economics describes a crowding out phenomenon in which increasing extrinsic motivation, such as financial incentives, reduces intrinsic motivation to act on one’s own [56]. There is limited evidence that motivation crowding out occurs in health-related behaviors [57], but this effect is difficult to evaluate and has not been previously studied with respect to various levels of financial incentives for smoking cessation to our knowledge.

Seventy-nine percent of the respondents stated an interest in smoking cessation prior to completing the DCE. A previous study at our institutions found that 48% of smokers that sustained a traumatic orthopaedic injury stated that their injury made them more likely to quit [58]. If we assume the 35% of eligible participants that refused to participate in the DCE also had limited interest in smoking cessation, the findings are relatively consistent. However, the link between an injury and the willingness to quit smoking requires further exploration. The time of injury may represent an opportunity for attitudinal change and associated with a shift in the valuation of habits and well-being [59–62].

Research suggests that smoking is more than twice as prevalent in the orthopaedic trauma population than the general US population, with rates approximating 50% compared to 20%, respectively [63, 64]. The epidemiological burden of smoking in orthopaedic trauma patients may be further exasperated by socioeconomic conditions. Our sample and the broader literature indicate the smoking population has a generally lower socioeconomic status [64, 65]. The median annual household income in our sample was $27,500. This measure is considerably lower than the median income for the US State of Maryland, where the study was conducted, which stands at $83,242 according to the 2018 American Community Survey [66]. Data from the US Department of Health and Human Services and the Administration of Substance Abuse and Mental Health Services supports this finding, noting that smoking is more common in individuals living below the poverty level [64, 65]. While we did not observe heterogeneity in preferences for financial incentives based on pre-injury household income, we also had few respondents with pre-injury household incomes above the state median to adequately test heterogeneity on this covariate. However, consistency in intervention effect across various demographic characteristics was also observed in another trial providing financial incentives to quit smoking [38]. The evaluation of financial incentive programs in patients after an orthopaedic injury is required to assess if these stated preferences remain robust across various demographic and socioeconomic characteristics.

Thirty-five percent of eligible participants declined to complete the survey. This rate of refusal could be attributed to disinterest in research, inconvenience, or resistance to quitting. Of note, we did not compensate respondents for completing the survey, and participant interest may be higher for an actual financial incentive program. In the study, 15% of respondents indicated an unwillingness to quit smoking regardless of the benefits from a financial incentive or protection against postoperative complications.

To our knowledge, this study is the first to investigate orthopaedic trauma patient preferences for financial incentives to stop smoking. The strengths of this study include actionable findings for smoking cessation programs or the design for future trials. The sample size was sufficient for precise utility estimates. The characteristics of the patient population were similar to prior smoking cessation studies in orthopaedic trauma patients [7, 16].

The study had several limitations. Patients consented for the survey in their postoperative follow-up appointments, and the results may not be consistent with preferences immediately after injury. Variation in the timing of the proposed payment was not assessed as part of this study. A recent clinical trial suggests the combination of a prepaid and promised incentive to be most efficient in increasing smoking quit rates [67]. Future research considers how to optimize the timing of financial incentives relative to injury. Complications experienced by patients may affect their preferences for payment and willingness to quit smoking. This study did not analyze preferences based on complication occurrence, but we recommend future research address this. We did not observe substantial variation in preferences based on characteristics, such as sex, race, education, income, and neighborhood deprivation. These null findings may be due to our limited sample and a narrow distribution of covariates, such as income, within the sample. Our study was performed at one center, so the generalizability of our findings to other patient groups is unknown and awaits confirming research. Finally, we did not measure the intrinsic motivation of the patients to quit smoking and are unable to assess the impact of that covariate on our estimates.

Our data suggest that orthopaedic trauma patients are willing to quit smoking during recovery for a financial incentive. These findings are consistent with those reported in the literature [34.38]. It is possible that in the short-term, extrinsic motivation to stop smoking coming from monetary payment does not crowd out intrinsic effort. In the long-run, habits and lack of self-control may cause relapse in these patients and reduce the effect of monetary rewards.

## Conclusions

Financial incentives may be an effective tool to promote smoking cessation in the orthopaedic trauma population if proven cost-effective. More research on the cost-effectiveness of financial incentive programs to prevent harmful health conditions is needed to justify policies and third-party funding for incentive programs [68]. The findings of our study define optimal payment thresholds for smoking cessation programs in a US study population. In the context of patient-centered research, it is central that we consider patient preferences when implementing smoking cessation interventions to improve intake and acceptance. Financial incentive programs to stop smoking in the short-term hold a promise to improve outcomes and reduce cost in the orthopaedic trauma population. Future studies applying financial incentives to promote smoking cessation should integrate patient preferences in the design and implementation of these programs.

## Data Availability

The study data will be made available upon written request to the corresponding author.

## Appendix

**Table 2:** Table of attributes and levels included in the discrete choice experiment

Attribute	Levels
Financial incentive	$0, $200, $400, $600, $800, $1000
**Nonunion:** Risk of a bone healing issue needing another surgery	3, 5, 8, 10%
**Deep infection:** Risk of a bone infection needing one or more surgeries to clean out infection, followed by 6 weeks of antibiotics	3, 6, 11, 15%
**Superficial infection:** Risk of a skin infection needing antibiotic pills for 2 weeks	5, 10, 15, 20%
